# Associations between cognitive and personality traits and work productivity loss in desk workers with low back pain: a cross-sectional study

**DOI:** 10.1539/eohp.2025-0025

**Published:** 2026-04-16

**Authors:** Takahiro Miki, Ryo Shiraishi, Yuta Hagiwara

**Affiliations:** 1 Insight Lab, PREVENT Inc., Aichi, Japan; 2 Graduate School, Saitama Prefectural University, Saitama, Japan

**Keywords:** cognitive beliefs, low back pain, personality traits, presenteeism, psychological factors, work productivity

## Abstract

**Objectives:**

This study investigated the associations between cognitive and personality traits and work productivity loss among desk workers with low back pain (LBP).

**Methods:**

A cross-sectional study was conducted among 2,223 corporate employees in Japan. Participants completed an online questionnaire assessing work productivity (quantity-quality method), pain intensity (numerical rating scale), disability (Roland-Morris Disability Questionnaire), beliefs about LBP (Back Beliefs Questionnaire), and personality traits (Ten Item Personality Inventory). Multiple linear regression analysis examined factors associated with productivity loss. To confirm robustness, a sensitivity analysis was performed limited to participants who identified LBP as the primary cause of their productivity loss.

**Results:**

Among the participants, 1,201 (54.0%) reported LBP. Regression analysis indicated that beliefs about LBP (β=−0.40, p=0.011), conscientiousness (β=−0.99, p=0.006), neuroticism (β=1.02, p=0.007), and age (β=−0.18, p=0.002) were significantly associated with work productivity loss. Pain intensity (p=0.139) and functional disability (p=0.574) were not significantly associated with productivity loss. These findings were consistent in the sensitivity analysis, where pain intensity remained unrelated to productivity loss.

**Conclusions:**

Psychological and cognitive factors, particularly beliefs about LBP and personality traits, may have a stronger impact on work productivity than physical symptoms among desk workers. Interventions focusing on cognitive restructuring and personality-tailored strategies are necessary to mitigate productivity losses.

## Introduction

Work productivity costs arise when illness affects individual performance, manifesting as both absenteeism (absence from work) and presenteeism (reduced performance while at work)^1)^. In particular, presenteeism represents a significant portion of productivity loss, as employees are physically present but unable to function at full capacity due to health issues. Musculoskeletal disorders contribute significantly to these costs, with up to 15% of affected individuals retiring early due to health-related reasons^2,3,4)^.

Low back pain (LBP) is a prevalent health issue and the primary cause of years lived with disability globally^5)^. While its direct mortality impact is minimal, it generates substantial medical and non-medical expenses for individuals, employers, and healthcare systems^6,7)^. With global population aging, these financial burdens are expected to increase. Indirect costs from lost or reduced work productivity are the largest contributor to LBP’s total economic impact, exceeding direct healthcare costs^8)^. In the United States, back and neck pain accounted for 264 million lost workdays annually, leading to an estimated income loss of ＄131.8 billion^9)^. Studies consistently show that back pain is significantly associated with increased presenteeism and work impairment^10)^.

While LBP is often viewed as a physical disorder, psychological and cognitive factors — such as fear-avoidance beliefs, pain catastrophizing, and low self-efficacy — have been shown to influence pain perception, disability, and work performance^11,12,13)^. Emerging evidence highlights the crucial role of these psychosocial factors in pain perception and disability^14)^. Studies indicate that these factors also contribute to productivity loss, particularly presenteeism^15,16)^. Additionally, individual personality traits may influence the development and persistence of musculoskeletal conditions, including LBP^17,18)^.

Despite extensive research on LBP’s impact on work productivity, the mechanisms underlying presenteeism remain insufficiently explored. While physical symptoms contribute to work limitations, psychological and cognitive factors may play equally significant roles in shaping work performance. Individual personality traits, such as conscientiousness and neuroticism, may influence how employees perceive and manage their pain, further affecting productivity. Understanding these factors is essential for developing effective workplace interventions that address not only physical impairments but also psychological barriers and personality-driven differences. We focused specifically on desk workers because prolonged sedentary behavior is a well-established risk factor for LBP, and restricting the study to this group minimizes the confounding effects of heavy physical workload commonly found in manual labor.

Given these gaps in the literature, this study aims to clarify the relationship between LBP and work productivity among desk workers, with particular focus on how psychological factors and personality traits influence work productivity.

## Methods

### Study design and participants

This cross-sectional study investigated the relationship between LBP, productivity, and related health factors among corporate employees in Japan. An online questionnaire was distributed to all 3,953 employees of the participating companies via the corporate health management system between October and November 2024. Participants without LBP were included to serve as a comparison group for evaluating the relative impact of LBP on productivity and economic costs. Participants were full-time employees, including those in managerial positions, working for a private insurance company with offices in major urban areas of Japan. They were primarily engaged in standard daytime work (8 hours/day). Survey responses were anonymized and stored securely. Participants with incomplete responses were excluded from analysis.

### Ethics approval

This study was performed in line with the principles of the Declaration of Helsinki and approval was granted by the Ethics Committee of PREVENT Inc. Informed consent was obtained from all individual participants included in the study.

### Measures

#### Demographic and occupational characteristics

Participants reported their gender, age, height, weight, and workplace location. Job-related information, including job type (eg, desk work, fieldwork), was also collected.

#### Labor productivity

The calculation of productivity loss using the Quantity-Quality (QQ) method was performed as follows: labor productivity was assessed using the QQ method, a validated tool for evaluating presenteeism^19)^. Participants self-reported their productivity as a percentage of their capacity under optimal health conditions. The QQ method consists of quantity and quality scales rated on a 0-10 numerical rating scale (NRS). Productivity loss was calculated as:

Productivity Loss Ratio = 1 – (Work Quantity (0-10)× Work Quality (0-10)) / 100

Participants reported health issues over the past 3 months and specified conditions most affecting work performance. Productivity loss was converted into monetary values using estimated average wages for economic impact assessment. The construct validity of this presenteeism measurement approach has been previously established^19)^.

#### Personality traits

Personality traits were evaluated using the Ten Item Personality Inventory (TIPI), assessing Big Five personality dimensions: Extraversion, Agreeableness, Conscientiousness, Emotional Stability, and Openness to Experience^20)^. Participants rated items on a 7-point Likert scale (1=Disagree strongly to 7=Agree strongly).

#### Physical activity level

Physical activity was assessed using the International Physical Activity Questionnaire (IPAQ), categorizing participants into low, moderate, or high activity levels based on weekly metabolic equivalent task (MET) minutes^21)^.

#### Low back pain assessment

LBP was assessed through self-reported binary response (Yes/No). Participants with LBP completed additional validated measures:

● Pain Severity: NRS (0=no pain to 10=worst pain imaginable)^22)^

● Disability: Roland Morris Disability Questionnaire (RMDQ), 24 items reflecting daily activities affected by back pain^23)^

● Beliefs: Back Belief Questionnaire (BBQ), 9 selected items rated on 5-point Likert scale (1=Completely Disagree to 5=Completely Agree). Higher scores indicate more positive beliefs about back pain prognosis^15)^.

### Data analysis

Descriptive statistics were computed for all variables. Group comparisons between participants with and without LBP used t-tests for continuous variables and chi-square tests for categorical variables. Multiple linear regression analysis examined factors influencing productivity loss among LBP participants. Independent variables included pain intensity (NRS), disability (RMDQ), physical activity (MET-min/week), beliefs (BBQ 9-item total), personality traits (extraversion, agreeableness, conscientiousness, neuroticism, openness), age, and gender. Multicollinearity was assessed using variance inflation factor (VIF; <10).

To confirm the robustness of our findings, we conducted a sensitivity analysis limited to participants who explicitly identified LBP as the primary cause of their work productivity loss. Statistical analyses used R software (R Foundation for Statistical Computing, Vienna, Austria) with significance set at p<0.05.

## Results

### Participant characteristics and group comparison

**Fig. 1.**
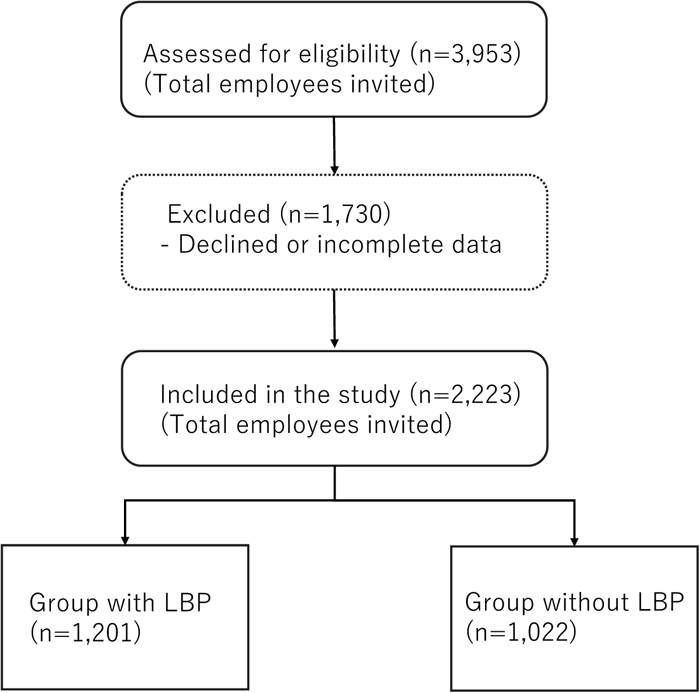
The participant selection process

Figure 1 illustrates the participant selection process. Of the 3,953 employees invited to participate, 2,223 completed the questionnaire (response rate: 56.2%). All 2,223 responses were complete and included in the final analysis. The mean age was 42.9 years (standard deviation [SD], 15.1), and average work productivity was 50.3% (SD, 26.0). Among participants, 1,201 (54.0%) reported experiencing LBP, while 1,022 (46.0%) did not. Participants with LBP were significantly older (43.8 vs. 41.5 years, p<0.001) compared to those without LBP. Although the LBP group reported slightly lower productivity (47.6% vs. 52.1%), this difference was not statistically significant (p=0.163). Among individuals with LBP, mean pain intensity (NRS) was 2.23 (SD, 2.24), mean disability (RDQ) score was 2.22 (SD, 3.74), and mean BBQ 9-item total score was 26.4 (SD, 5.7). Detailed characteristics are summarized in Table 1. Among the participants with LBP, 504 (42.0%) identified LBP as the health condition most affecting their work performance, whereas 697 (58.0%) attributed their productivity loss primarily to other health issues, such as neck/shoulder pain and lack of sleep (eTable 1).

**Table 1.** Characteristics of participant and Comparison between Groups with and without Low Back Pain

	Overall (n=2,223)	Group with low back pain (n=1,201)	Group without low back pain (n=1,022)	p value
Age, years, mean (SD)	42.9 (15.1)	44.5 (14.5)	40.6 (15.7)	<0.001
Gender, %				0.596
- Male	787 (35.4)	438 (36.5)	349 (34.1)	
- Female	1,404 (63.2)	744 (61.9)	660 (64.6)	
- No response	32 (1.4)	19 (2.6)	13 (1.3)	
Height, mean (SD)	160.6 (27.1)	160.9 (27.1)	160.3 (27.0)	0.714
Weight, mean (SD)	58.9 (18.6)	59.1 (18.5)	58.7 (18.6)	0.733
Work productivity, %, mean (SD)	50.3 (26.0)	49.6 (25.2)	51.2 (26.9)	0.163
Work productivity loss, %, mean (SD)	49.7 (26.0)	50.4 (25.2)	48.8 (26.9)	0.163
Personality traits				
- Extraversion, mean (SD)	8.1 (2.6)	8.2 (2.6)	8.0 (2.6)	0.035
- Agreeableness, mean (SD)	10.3 (1.9)	10.3 (1.9)	10.3 (1.9)	0.371
- Conscientiousness, mean (SD)	8.3 (2.3)	8.3 (2.4)	8.3 (2.3)	0.865
- Neuroticism, mean (SD)	7.9 (2.4)	8.0 (2.4)	7.9 (2.4)	0.216
- Openness, mean (SD)	7.6 (2.4)	7.7 (2.4)	7.5 (2.4)	0.113
Total physical activity, MET-min/week, mean (SD)	2,241.3 (10,270.2)	2,088.7 (9,833.5)	2,420.7 (10,762.9)	0.451
Physical activity category, %				
- Low	833 (37.5)	0.36 (0.48)	0.39 (0.49)	0.291
- Moderate	1,166 (52.5)	0.55 (0.50)	0.50 (0.50)	0.026
- High	224 (10.1)	0.09 (0.28)	0.11 (0.32)	0.050
Pain intensity, mean (SD)	—	2.2 (2.2)	—	—
Time since LBP onset, mean (SD)	—	64.7 (425.9)	—	—
RDQ, mean (SD)	—	1.2 (3.0)	—	—
BBQ total score	—	43.2 (7.1)	—	—
BBQ 9-item total score	—	26.4 (5.7)	—	—

BBQ, Back Belief Questionnaire; JPY, Japanese yen; MET, metabolic equivalent; RDQ, Roland Morris Disability Questionnaire; SD, standard deviation.

### Regression analysis

Multiple linear regression analysis examined factors associated with work productivity loss among individuals with LBP. Independent variables included pain intensity, disability, beliefs about LBP, physical activity, personality traits, age, and sex. Results indicated that beliefs about LBP (β=−0.40; 95% confidence interval [CI],−0.70 to−0.09, p=0.011), conscientiousness (β=−0.99; 95% CI,−1.69 to−0.28, p=0.006), neuroticism (β=1.02; 95% CI, 0.29 to 1.75, p=0.007), and age (β=−0.18; 95% CI,−0.30 to−0.06, p=0.002) were significantly associated with work productivity loss. More positive beliefs about LBP, higher conscientiousness, and older age were linked to lower productivity loss, whereas higher neuroticism was associated with greater productivity loss. Pain intensity (p=0.139), disability (p=0.574), physical activity (p=0.968), extraversion (p=0.919), agreeableness (p=0.342), and openness (p=0.397) were not significantly associated with productivity loss. The overall model accounted for 7.6% of the variance in work productivity loss (R^2^=0.076). VIF analysis confirmed all predictor variables had VIF<10, indicating no multicollinearity concerns. Key findings are summarized in Table 2. In the sensitivity analysis limited to participants who identified LBP as the primary cause of productivity loss, the results were largely consistent with the primary analysis (eTable 2). Beliefs about LBP and neuroticism remained significantly associated with work productivity loss, whereas pain intensity was not associated with productivity loss.

**Table 2.** Multiple linear regression analysis for work productivity loss among individuals with low back pain

Variable	β	95% CI		p-value
		Lower	Upper	
Pain intensity (NRS)	0.60	− 0.195	1.390	0.139
Low back pain disability (RDQ)	0.13	− 0.329	0.592	0.574
Beliefs about low back pain (BBQ 9-item total)	− 0.40	− 0.703	− 0.092	0.011
Physical activity (MET-min/week)	0.00	− 0.000	0.000	0.968
Extraversion	0.04	− 0.660	0.732	0.919
Agreeableness	− 0.42	− 1.300	0.451	0.342
Conscientiousness	− 0.99	− 1.692	− 0.280	0.006
Neuroticism	1.02	0.285	1.749	0.007
Openness	0.33	− 0.438	1.104	0.397
Age	− 0.18	− 0.296	− 0.064	0.002
Sex (Female = 1)	− 7.36	− 54.96	40.25	0.762
Constant	77.35	26.08	128.61	0.003

BBQ, Back Belief Questionnaire; CI, confidence interval; MET, metabolic equivalent of task; NRS, numerical rating scale; RDQ, Roland Morris Disability Questionnaire.

Adjusted R squared model (R^2^)=0.076.

## Discussion

This study examined associations between cognitive and personality traits and work productivity loss among employees with LBP. Results indicated that beliefs about LBP, conscientiousness, neuroticism, and age were significantly associated with productivity loss, while pain intensity and functional disability were not. These findings suggest that psychological and cognitive factors may have stronger impacts on productivity than physical symptoms among employees with LBP.

Our findings align with previous research demonstrating that negative beliefs about LBP are associated with increased disability and chronic pain development^16)^. Turner et al. reported that workers with high work-related fear-avoidance and low recovery expectations were significantly more likely to remain on disability at 6 months, even after adjusting for pain intensity and physical disability^24)^. Similarly, Fritz & George found that individuals with higher fear-avoidance beliefs exhibited greater difficulty returning to work following acute work-related LBP^25)^. These results support our finding that cognitive factors, rather than pain severity, were more strongly associated with productivity loss. Negative beliefs about LBP may lead to productivity loss through multiple pathways. Fear of worsening pain can result in avoidance behaviors, restricting work performance and reducing efficiency^26)^. Additionally, heightened pain awareness may consume cognitive resources, leading to reduced concentration and impaired decision-making^27)^. Lower self-efficacy regarding pain management may further exacerbate work impairment by decreasing motivation and engagement in work tasks^12)^.

This study also revealed that higher conscientiousness was associated with lower productivity loss, while higher neuroticism was linked to greater productivity loss. While previous research has examined the relationship between personality traits and job satisfaction, few studies have specifically investigated their role in work productivity loss among individuals with LBP. Neuroticism consistently has a negative association with all aspects of job satisfaction, whereas agreeableness and conscientiousness have positive associations with job satisfaction^28)^. Additionally, conscientiousness has been linked to better job performance^29)^. Another study reported that personality traits influence self-reported productivity, with conscientious individuals potentially attending work despite illness but acknowledging reduced productivity. Conversely, individuals with excessive work commitment may attend work but underestimate productivity declines^30)^. Among LBP populations, neuroticism is associated with higher levels of fear-avoidance, depression, and anxiety^17)^. Another study suggested that personality traits may influence pain perception and coping mechanisms^31)^. Our findings support the notion that neuroticism contributes to productivity loss by increasing emotional distress and maladaptive coping strategies, such as excessive worrying and avoidance behaviors^32)^. On the other hand, conscientious individuals, who are more structured and goal-oriented, tend to maintain productivity despite physical discomfort^33)^. Additionally, they may underreport presenteeism due to a strong work ethic, potentially leading to an underestimation of productivity loss in self-reported measures^30)^.

Pain intensity and functional disability were not significantly associated with work productivity loss in our study. This finding contrasts with some studies that emphasize the direct impact of pain severity on work performance. For instance, a study found that workers experiencing severe pain symptoms had higher probabilities of extended benefit durations, increased lost-earnings costs, and elevated healthcare expenditures compared to those without pain symptoms^34)^. Similarly, reducing pain severity has been linked to improvements in quality of life and work productivity^35)^. One possible explanation for these discrepancies is that the participants in this study reported relatively mild pain and functional impairment, limiting their impact on productivity loss. Additionally, individual differences in pain perception and coping strategies may explain these findings. Some individuals may experience substantial work impairment due to pain, while others may adopt adaptive coping strategies, such as cognitive reframing or pacing techniques, allowing them to maintain productivity despite discomfort. Psychological factors may mediate the relationship between pain and productivity. Martinez-Calderon et al. ound that positive psychological factors such as pain acceptance, self-efficacy, and optimism mediate the relationship between pain intensity and interference with daily activities^16)^. This suggests that individuals with higher levels of these positive psychological traits experienced less disruption in their daily activities despite similar levels of pain intensity. Furthermore, a systematic review highlighted that psychological factors, including pain-related worrying, optimism, and anxiety, contribute to the development and maintenance of chronic pain and disability^36)^. This underscores the significant role that cognitive perceptions and emotional responses play in the experience of pain and its impact on daily life. These findings suggest that interventions targeting psychological and cognitive factors may be more effective in mitigating the impact of pain on productivity than approaches solely focusing on pain reduction.

This study has several limitations. First, its cross-sectional design limits the ability to infer causal relationships between psychological factors, personality traits, and work productivity. The bidirectional nature of these relationships cannot be ruled out. Additionally, unmeasured confounding factors, such as specific workplace environments or job stress, may have influenced the results. Second, potential biases regarding participant selection must be considered. The response rate was 56.2%, and non-respondents may differ from respondents in key characteristics. For instance, employees with severe LBP might be on sick leave (absenteeism) and thus underrepresented in this study (healthy worker effect). This selection bias could lead to an underestimation of the true severity of LBP and its impact on productivity within the overall workforce. Third, all variables were assessed using self-reported measures, introducing potential common method bias. This is particularly relevant for the association between personality traits and self-rated productivity. Individuals with high neuroticism may perceive both their health and performance more negatively, potentially inflating the observed associations. Conversely, conscientious individuals may underreport productivity impairment due to excessive work commitment. However, the distinct patterns of association observed — where conscientiousness was protective while neuroticism was detrimental — suggest that the findings reflect meaningful relationships rather than solely reporting artifacts. Fourth, the study sample consisted solely of desk-based employees in a private insurance company, limiting generalizability to other occupational groups or industries. Detailed employment characteristics could not be fully disclosed due to confidentiality agreements. Future studies should examine diverse occupations, incorporate longitudinal designs, and use objective productivity measures.

### Clinical implications and future directions

The findings underscore the importance of adopting a holistic perspective in occupational health management that considers not only productivity metrics but also the psychological well-being of employees. For example, occupational health specialists could utilize brief screening tools to help employees recognize their own personality traits, such as high neuroticism, excessive work commitment, and cognitive biases. Increasing self-awareness regarding these traits may help employees understand how presenteeism exacerbates physical and emotional strain, thereby preventing future productivity loss and promoting long-term health.

### Conclusion

This study highlights the significant role of psychological and cognitive factors, particularly beliefs about LBP and personality traits, in influencing work productivity loss among employees with LBP. Negative beliefs about LBP and higher neuroticism were associated with greater productivity loss, whereas higher conscientiousness was linked to lower productivity loss. In contrast, pain intensity and functional disability did not show significant associations with productivity loss, suggesting that psychological and cognitive factors may have a stronger impact on work performance. These findings emphasize the need for interventions focusing on cognitive restructuring, stress management, and personality-tailored strategies to mitigate productivity losses. Future longitudinal research incorporating objective productivity measures is necessary to clarify causal relationships and optimize workplace interventions for employees with LBP.

## Supplementary Material

Supplementary eTable 1

Supplementary eTable 2

## Data Availability

The datasets generated and/or analyzed during the current study are available from the corresponding author upon reasonable request.
