# Supplementary material for: Associations between cognitive and personality traits and work productivity loss in desk workers with low back pain: a cross-sectional study
Source: Environ Occup Health Pract. 2026 Apr 16;8(1):2025-0025. doi: 10.1539/eohp.2025-0025 (PMC13293773; doi:10.1539/eohp.2025-0025)
Supplement: Supplementary file 1 — Supplementary eTable 1 [file eohp-8-2025-0025-s001.pdf]

eTable 1. Primary causes of work productivity loss among participants with low back pain

Primary cause of work productivity loss	n	%
Low back pain	504	42
Other health conditions	697	58
Breakdown of other conditions (Top 5)		
Stiff shoulders / Neck pain	327	27.2
Lack of sleep	223	18.6
Headache	177	14.7
Eye strain / Eye disease	139	11.6
Fatigue	66	5.5
