# Supplementary material for: Associations between cognitive and personality traits and work productivity loss in desk workers with low back pain: a cross-sectional study
Source: Environ Occup Health Pract. 2026 Apr 16;8(1):2025-0025. doi: 10.1539/eohp.2025-0025 (PMC13293773; doi:10.1539/eohp.2025-0025)
Supplement: Supplementary file 2 — Supplementary eTable 2 [file eohp-8-2025-0025-s002.pdf]

eTable 2. Sensitivity analysis: Multiple linear regression analysis for work productivity loss among participants who identified low back pain as the primary cause of productivity loss

Variable	*β*	95% CI	p-value
Pain intensity (NRS)	0.01	−1.12 to 1.13	0.99
Low back pain disability (RDQ)	0.17	−0.43 to 0.76	0.579
Beliefs about low back pain (BBQ)	-0.59	−1.01 to −0.17	0.006
Physical activity (MET-min/week)	0	0.00–0.00	0.104
Extraversion	0.57	−0.46 to 1.59	0.275
Agreeableness	-0.63	−1.84 to 0.58	0.309
Conscientiousness	-0.83	−1.81 to 0.14	0.093
Neuroticism	1.67	0.57–2.76	0.003
Openness	0.51	−0.62 to 1.63	0.379
Age	-0.2	−0.35 to −0.05	0.008
Sex (Female = 1)	-4.38	−9.31 to 0.55	0.082

BBQ, Back Beliefs Questionnaire; CI, confidence interval; MET, metabolic equivalent of task; NRS, numerical rating scale; RDQ, Roland-Morris Disability Questionnaire.

Note: Bold values indicate statistical significance ($p < 0.05$).
